# Rolled Dielectric Elastomer Antagonistic Actuators for Biomimetic Underwater Robots

**DOI:** 10.3390/polym14214549

**Published:** 2022-10-27

**Authors:** Toshiaki Nagai, Jun Shintake

**Affiliations:** Department of Mechanical and Intelligent Systems Engineering, Graduate School of Informatics and Engineering, The University of Electro-Communications, 1-5-1 Chofu-gaoka, Chofu, Tokyo 182-8585, Japan

**Keywords:** dielectric elastomer actuators, soft robotics, underwater robots, biomimetic robots

## Abstract

In this study, an antagonistic actuator using dielectric elastomer actuators (DEAs) is developed to investigate the use of rolled DEAs in underwater robots. The actuator consists of a backbone, an elastic hinge, and two rolled DEAs placed in an antagonistic fashion, allowing for the generation of bidirectional movements of the actuator tip. To prove this concept, an analytical model of the actuator is built. The experimental samples are fabricated based on the specification determined by the model. In the fabricated actuator, each rolled DEA has a diameter of 6 mm and a length of 21 mm. The whole device weighs 1.7 g. In the tested voltage range of 0–1200 V, the actuator exhibits a voltage-controllable angle and torque of up to 2.2° and 11.3 mN∙mm, respectively. The actuator is then implemented into a swimming robot, which shows forward speed of 0.9 mm/s at the applied voltage of 1000 V and the driving frequency of 10 Hz. The results demonstrate the feasibility of using rolled DEAs in underwater robots.

## 1. Introduction

Dielectric Elastomer Actuators (DEAs) are a promising soft actuator technology in the field of soft robotics [1,2,3,4], which has received a significant research effort in recent years [5,6,7,8]. DEAs are a type of electrostatic actuator that work by converting electrical energy into mechanical energy. They are usually composed of an elastomeric membrane and two compliant electrodes placed on both sides of the membrane. Under application of a high voltage (a few kV), the electrostatic force between the electrodes squeezes the membrane in the thickness direction and expands it in the planar direction. The features of DEAs are represented as: high compliance, large active deformation, fast response, and high energy density [9,10,11]. Moreover, their characteristics are similar to those of mammalian muscles [11].

As an application of DEAs, biomimetic underwater robots have been a topic receiving much research effort. The reason behind this is that the features of DEAs, such as compliance and large deformation, are thought to be suitable for mimicking the structures and swimming behavior of fish leading to a realization of advanced underwater systems. Moreover, the simplicity of the structure allows applying DEAs to diverse morphologies. In these contexts, researchers have developed DEA-based biomimetic underwater robots in different forms such as fish [12,13], jellyfish [14,15,16,17,18], ray [19], eel [20,21], cephalopod [22], and frog [23].

In DEA-based swimming robots, the configuration of the actuator plays an important role in determining the resulting locomotion characteristics. As a configuration of DEAs, the rolled type has several features, which are expected to be effective for the underwater robots. This type of DEA configuration exhibits muscle-like linear actuation and is relatively easy to perform scaling when compared to other configurations [24,25,26,27]. However, to the best of our knowledge, no study is conducted on the use of rolled DEAs for underwater robots.

**Figure 1:**
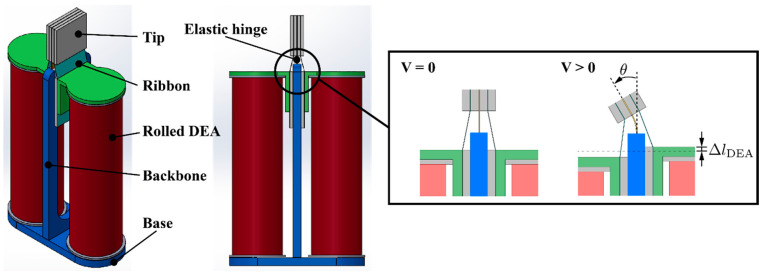
Structure and working principle of the antagonistic actuator proposed in this study.

The aim of this study is to investigate the use of rolled DEAs for underwater robots. For this purpose, we propose and develop an antagonistic actuator shown in Figure 1. It has a rigid backbone, an elastic hinge, and two rolled DEAs placed in an antagonistic fashion. The entire architecture of the actuator is inspired by the endoskeletal structures of fish, where muscles are placed across a backbone. In the actuator, the rolled DEAs are pre-stretched so that the tip is centered under no voltage. When a voltage is applied to one of these DEAs, the activated part extends while the other one shrinks, resulting in a bending deformation of the hinge; therefore, an angular displacement of the tip. Alternate actuation of each DEA allows for bidirectional movements, which we assume are suitable for underwater locomotion, given many fish swim with symmetric deformation of a fin.

In the rest of this paper, we first construct an analytical model of the actuator. After that, we fabricate and characterize the actuator samples to clarify their actuation behaviors. Subsequently, we develop a fish-type underwater robot and test it in a water environment to demonstrate the applicability of the proposed actuator. Finally, we discuss and conclude the results. 

## 2. Working Principle and Model

As mentioned previously, the actuator has two rolled DEAs. One side of the DEAs are fixed to the base part, and the other side is connected to the tip via a ribbon. In the actuator, the DEAs are pre-stretched. The backbone and tip are connected with an elastic hinge. When a voltage is applied to one of the DEAs, it is elongated while the other one is contracted. This causes the elastic hinge to bend, which in turn rotates the tip.

In order to predict the behavior of the actuator for the design purpose, we built an analytical model. In this model, the tip angle θ is calculated with respect to the applied voltage to the DEAs. θ is determined from the minimum value of the total potential energy in the actuator $U_{\mathrm{total}}$. The condition for minimizing $U_{\mathrm{total}}$ is,

(1) $$\frac{\partial U_{\mathrm{total}}}{\partial \theta }=0\;\mathrm{and}\;\frac{\partial^{2}U_{\mathrm{total}}}{\partial \theta^{2}}>0$$

$U_{\mathrm{total}}$ consists of the following terms,

(2) $$\begin{array}{c} U_{\mathrm{total}}=U_{{\mathrm{strain}}_{{\mathrm{DEA}}_{a}}}+U_{e{\mathrm{lectric}}_{{\mathrm{DEA}}_{a}}}\\ +U_{{\mathrm{strain}}_{{\mathrm{DEA}}_{b}}}+U_{{\mathrm{electric}}_{{\mathrm{DEA}}_{b}}}\\ +U_{\mathrm{ribbon}\_a}+U_{\mathrm{ribbon}\_b}+U_{\mathrm{hinge}} \end{array}$$

where $U_{\mathrm{strain}\_\mathrm{DEA}\_a}$ and $U_{\mathrm{strain}\_\mathrm{DEA}\_b}$ are the strain energy of the DEA on side a and b, respectively. Similarly, $U_{\mathrm{electric}\_\mathrm{DEA}\_a}$ and $U_{\mathrm{electric}\_\mathrm{DEA}\_b}$ are the electrostatic energy of the DEA on each side, and $U_{\mathrm{ribbon}\_a}$ and $U_{\mathrm{ribbon}\_b}$ are the elastic energy of the ribbons. $U_{\mathrm{hinge}}$ is the elastic energy of the hinge. In the model, each of these energies is derived as a function of θ.

**Figure 2:**
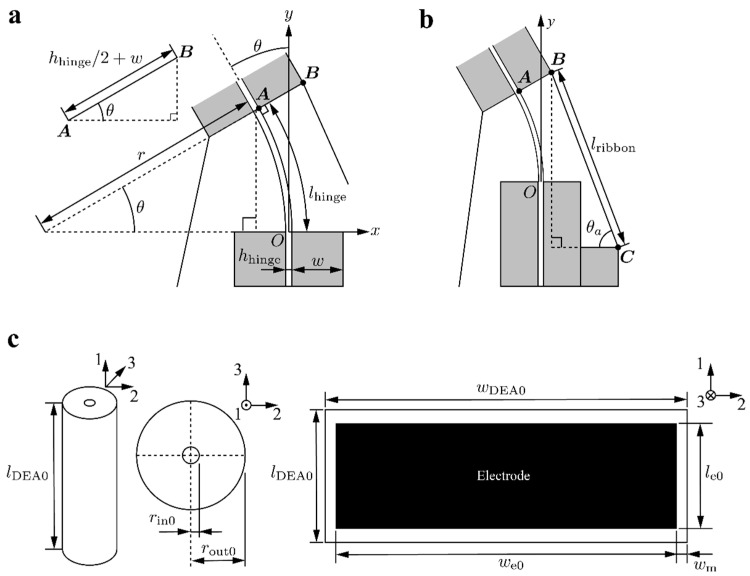
(**a**,**b**) Schematics of the joint part of the actuator used for the model. (**c**) Schematics of the rolled DEA used for the model.

In order to express the energies related to the DEAs, the coordinates of the points in the schematics, shown in Figure 2a,b are obtained. When the actuator generates a tip angle θ, the following relationship can be established between the radius of curvature r and the length of the hinge $l_{\mathrm{hinge}}$.

(3) $$r\theta =l_{\mathrm{hinge}}$$

Using the auxiliary line shown in Figure 2a, the coordinates of point A is expressed as:

(4) $$A=\left[\begin{array}{c} -r\left(1-\cos\theta \right)\\ r\sin\theta \end{array}\right]=\left[\begin{array}{c} -\frac{l_{\mathrm{hinge}}}{\theta }\left(1-\cos\theta \right)\\ \frac{l_{\mathrm{hinge}}}{\theta }\sin\theta \end{array}\right]$$

The coordinates of point B can be obtained using the thickness of the hinge $h_{\mathrm{hinge}}$ and the width of the fixing parts w as follows:

(5) $$\begin{array}{c} B|=A+\left[\begin{array}{c} \left(\frac{h_{\mathrm{hinge}}}{2}+w\right)\cos\theta \\ \left(\frac{h_{\mathrm{hinge}}}{2}+w\right)\sin\theta \end{array}\right]\\ |=\left[\begin{array}{c} -\frac{l_{\mathrm{hinge}}}{\theta }\left(1-\cos\theta \right)+\left(\frac{h_{\mathrm{hinge}}}{2}+w\right)\cos\theta \\ \frac{l_{\mathrm{hinge}}}{\theta }\sin\theta +\left(\frac{h_{\mathrm{hinge}}}{2}+w\right)\sin\theta \end{array}\right] \end{array}$$

Then, based on the auxiliary line represented in Figure 2b, the angle of ribbon $\theta_{a}$ can be expressed by using its length $l_{\mathrm{ribbon}}$:

(6) $$\theta_{a}=\cos^{-1}\left(\frac{\frac{h_{\mathrm{hinge}}}{2}+2w-Bx}{l_{\mathrm{ribbon}}}\right)$$

The coordinates of point C become,

(7) $$C=\left[\begin{array}{c} 2w\\ \left(\frac{l_{\mathrm{hinge}}}{\theta }+\frac{h_{\mathrm{hinge}}}{2}+w\right)\sin\theta -l_{\mathrm{ribbon}}\sin\theta_{a} \end{array}\right]$$

Given that the initial angle is θ=0, $\theta_{a0}$ is given as,

(8) $$\theta_{a0}=\cos^{-1}\left(\frac{w}{l_{\mathrm{ribbon}}}\right)$$

The initial position $C_{0}$ can be expressed as,

(9) $$C_{0}=\left[\begin{array}{c} 2w\\ l_{\mathrm{hinge}}-l_{\mathrm{ribbon}}\sin\theta_{a0} \end{array}\right]$$

The displacement $\Delta l_{\mathrm{DEA}}$ is then obtained as,

(10) $$\Delta l_{\mathrm{DEA}}=C_{y}-C_{y0}$$

The elastomer of the DEA is known to be incompressible and the following relationship can be established:

(11) $$\lambda_{1}\lambda_{2}\lambda_{3}=1$$

Here, $\lambda_{i}$ represents the stretch in the three directions illustrated in Figure 2c. Let $l_{\mathrm{DEA}0}$, $w_{\mathrm{DEA}0}$, and $h_{\mathrm{DEA}0}$ be the length, width, and thickness of the DEA before rolling, respectively. Similarly, let $l_{\mathrm{DEA}}$, $w_{\mathrm{DEA}}$, and $h_{\mathrm{DEA}}$ be their value in a deformation state. Using these parameters, the stretches of the DEA can be expressed as:

(12) $$\lambda_{1}=\frac{l_{\mathrm{DEA}}}{l_{\mathrm{DEA}0}},\;\lambda_{2}=\frac{w_{\mathrm{DEA}}}{w_{\mathrm{DEA}0}},\;\lambda_{3}=\frac{h_{\mathrm{DEA}}}{h_{\mathrm{DEA}0}}$$

In the model, the DEA is assumed to be a solid cylinder, meaning that the radial strains $\lambda_{r}$ are equal during deformation. This assumption is also verified in the literature for rolled DEAs [28]. The following relationship holds from Equation (11) as follows:

(13) $$\lambda_{1}\lambda_{2}\lambda_{3}=\lambda_{1}\lambda_{r}^{2}=1$$

From this, the radial strain $\lambda_{r}$ can be obtained as: $\lambda_{r}=1/\sqrt{\lambda_{1}}$. As mentioned previously, the DEAs are pre-stretched in the actuator. Based on the pre-stretch in the single-axis direction $\lambda_{1p}$, the radial pre-stretch $\lambda_{\mathrm{rp}}$ is given as: $\lambda_{\mathrm{rp}}=1/\sqrt{\lambda_{1p}}$. In addition, the dimensional parameters of the pre-stretched DEA are:

(14) $$\;l_{\mathrm{DEAp}}=\lambda_{1p}l_{\mathrm{DEA}0},\;\;w_{\mathrm{DEAp}}=\lambda_{\mathrm{rp}}w_{\mathrm{DEA}0},\;h_{\mathrm{DEAp}}=\lambda_{\mathrm{rp}}h_{\mathrm{DEA}0}\;$$

Consider that a voltage is applied to the DEA on side a, its displacement $\Delta l_{\mathrm{DEA}\_a}$ is:

(15) $$\Delta l_{\mathrm{DEA}\_a}=l_{\mathrm{DEA}\_a}-l_{\mathrm{DEA}\_\mathrm{ap}}$$

Here, the following relationship holds from Equation (10),

(16) $$l_{\mathrm{DEA}\_a}\left(\theta \right)=l_{\mathrm{DEA}\_\mathrm{ap}}+C_{y}\left(\theta \right)-C_{y0}$$

The stretch $\lambda_{1a}$ of the DEA on side a can be expressed as a function of θ from Equations (12) and (14) as follows:

(17) $$\lambda_{1a}\left(\theta \right)=\frac{l_{\mathrm{DEA}\_a}\left(\theta \right)}{l_{\mathrm{DEA}\_a0}}=\lambda_{1p}+\frac{C_{y}\left(\theta \right)-C_{y0}}{l_{\mathrm{DEA}\_a0}}$$

Since the stretch $\lambda_{1b}$ of the DEA on side b changes opposite to the other side, it is given as:

(18) $$\lambda_{1b}\left(\theta \right)=\lambda_{1p}-\frac{C_{y}\left(\theta \right)-C_{y0}}{l_{\mathrm{DEA}\_a0}}$$

To calculate the strain energy of DEAs, the Yeoh hyperelastic material model [29] is used, in which the strain energy density function W is given as follows:

(19) $$W=\overset{3}{\underset{i=1}{\sum }}C_{i}{\left(I_{1}-3\right)}^{i}$$

where $C_{i}$ is the material constant and $I_{1}=\lambda_{1}^{2}+\lambda_{2}^{2}+\lambda_{3}^{2}$. From Equations (17) and (18), the strain energy of $U_{\mathrm{strain}\_\mathrm{DEA}}$ can be expressed by the following equation:

(20) $$\begin{array}{c} \;U_{{\mathrm{strain}}_{\mathrm{DEA}}}\left(\theta \right)=vol\cdot \overset{3}{\underset{i=1}{\sum }}C_{i}{\left(I_{1}\left(\theta \right)-3\right)}^{i}\\ I_{1}\left(\theta \right)={\left\{\lambda_{1}\left(\theta \right)\right\}}^{2}+\frac{2}{\lambda_{1}\left(\theta \right)}\\ vol|=l_{\mathrm{DEA}0}w_{\mathrm{DEA}0}h_{\mathrm{DEA}0} \end{array}$$

Since the DEA has a capacitor-like structure, the electrostatic energy $U_{\mathrm{electric}\_\mathrm{DEA}}$ under an applied voltage V is:

(21) $$U_{\mathrm{electric}\_\mathrm{DEA}}=-\frac{1}{2}C_{\mathrm{DEA}}V^{2}=-\frac{1}{2}\varepsilon_{0}\varepsilon_{r}\frac{S_{e}}{d_{\mathrm{DEA}}}\;V^{2}$$

Here, $C_{\mathrm{DEA}}$ is the capacitance, $S_{e}$ the electrode area, $d_{\mathrm{DEA}}$, the distance between the electrodes, $\varepsilon_{0}$ the vacuum permittivity, and $\varepsilon_{r}$ is the dielectric constant of the elastomer. $S_{e}$ and $d_{\mathrm{DEA}}$ change depending on the strain of the DEA. The electrostatic energy is negative because the applied voltage is inputted from outside the DEA.

In the rolled DEA employed in this study, the inner and outer dielectric layers overlap to form a single dielectric layer. Since the polarity of the electrode layers on both sides is different, an electric field is generated in the overlapping dielectric layer and it functions as a DEA. Therefore, the electrode area of the DEA is twice the area of the electrode before the roll, minus the area of the electrodes on the inner and outer parts of the structure where no electric field is generated.

As shown in Figure 2c, consider a DEA with an initial length and width as $l_{e0}$ and $w_{e0}$, respectively, and a margin of $w_{m}$. After rolling, the DEA has a cylindrical shape with inner diameter $r_{\mathrm{in}0}$, outer diameter $r_{\mathrm{out}0}$, and height $l_{\mathrm{DEA}0}$. By using $\lambda_{1}$ and $\lambda_{2}$, the electrode area $S_{e0}$ can be expressed as:

(22) $$S_{e}=2\lambda_{1}\lambda_{2}l_{e0}\left\{w_{e0}-\pi \left(r_{\mathrm{in}0}+r_{\mathrm{out}0}\right)+w_{m}\right\}$$

The distance between the electrodes $d_{e}$ can be expressed as $d_{DEA}=\lambda_{3}d_{DEA0}$ using its initial value $d_{e0}$ and $\lambda_{3}$. Then, $U_{\mathrm{electric}\_\mathrm{DEA}}$ can be obtained as a function of θ from Equations (21) and (22) as follows:

(23) $$\begin{array}{c} U_{\mathrm{electric}\_\mathrm{DEA}}\left(\theta )\right|=-\frac{1}{2}\varepsilon_{0}\varepsilon_{r}\frac{S_{e}}{d_{e}}\;V^{2}\\ |=-\varepsilon_{0}\varepsilon_{r}\frac{\lambda_{1}\left(\theta \right)l_{e0}\left\{w_{e0}-\pi \left(r_{\mathrm{in}0}+r_{\mathrm{out}0}\right)+w_{m}\right\}}{d_{e0}}\;V^{2} \end{array}$$

From the volume vol of the DEA in Equation (20), the following relationship is established for the inner diameter $r_{\mathrm{in}0}$ and outer diameter $r_{\mathrm{out}0}$ of the DEA:

(24) $$vol=l_{\mathrm{DEA}0}\pi \left(r_{\mathrm{out}0}^{2}-r_{\mathrm{in}0}^{2}\right)=l_{\mathrm{DEA}0}w_{\mathrm{DEA}0}h_{\mathrm{DEA}0}$$

The elastic energies of the two ribbons and hinges, $U_{\mathrm{ribbon}\_i}$ and $U_{\mathrm{hinge}}$, are calculated using θ and the rotational spring constants $k_{\mathrm{ribbon}}^{\prime }$ and $k_{\mathrm{hinge}}^{\prime }$ for the ribbon and hinge, respectively, as follows:

(25) $$U_{\mathrm{ribbon}\_i}=\frac{1}{2}k_{\mathrm{ribbon}}^{\prime }\theta^{2},\;U_{\mathrm{hinge}}=\frac{1}{2}k_{\mathrm{hinge}}^{\prime }\theta^{2}$$

$k_{\mathrm{ribbon}}^{\prime }$ and $k_{\mathrm{hinge}}^{\prime }$ are determined by the material properties, dimensions, and shape of the ribbon and hinge as follows:

(26) $$\begin{array}{c} k_{\mathrm{ribbon}}^{\prime }|=\frac{E_{\mathrm{ribbon}}I_{\mathrm{ribbon}}}{l_{\mathrm{ribbon}}}=\frac{E_{\mathrm{ribbon}}w_{\mathrm{ribbon}}h_{\mathrm{ribbon}}^{3}}{12l_{\mathrm{ribbon}}}\\ k_{\mathrm{hinge}}^{\prime }|=\frac{E_{\mathrm{hinge}}I_{\mathrm{hinge}}}{l_{\mathrm{hinge}}}=\frac{E_{\mathrm{hinge}}w_{\mathrm{hinge}}h_{\mathrm{hinge}}^{3}}{12l_{\mathrm{hinge}}} \end{array}$$

where $E_{i}$ is the respective Young’s modulus of the ribbon and hinge, $I_{i}$ is the cross-sectional second moment, and $w_{i}$ and $h_{i}$ are the width and thickness, respectively.

From the above, the total potential energy in the system $U_{\mathrm{total}}$ can be expressed as a function of θ. Partial differentiation of $U_{\mathrm{total}}$ with respect to θ allows to find θ for a given applied voltage as follows:

(27) $$\begin{array}{c} \;\frac{\partial U_{\mathrm{total}}}{\partial \theta }|=\frac{\partial U_{{\mathrm{strain}}_{{\mathrm{DEA}}_{a}}}}{\partial \theta }+\frac{\partial U_{e{\mathrm{lectric}}_{{\mathrm{DEA}}_{a}}}}{\partial \theta }\\ +\frac{\partial U_{{\mathrm{strain}}_{{\mathrm{DEA}}_{b}}}}{\partial \theta }+\frac{\partial U_{e{\mathrm{lectric}}_{{\mathrm{DEA}}_{b}}}}{\partial \theta }\\ +\frac{\partial U_{\mathrm{ribbon}\_a}}{\partial \theta }+\frac{\partial U_{\mathrm{ribbon}\_b}}{\partial \theta }+\frac{\partial U_{\mathrm{hinge}}}{\partial \theta }|=0 \end{array}$$

Furthermore, from θ, $k_{\mathrm{ribbon}}^{\prime }$ and $k_{\mathrm{hinge}}^{\prime }$, the torque of the actuator τ can be obtained from the following equation:

(28) $$\tau =2k_{\mathrm{ribbon}}^{\prime }\theta +k_{\mathrm{hinge}}^{\prime }\theta$$

**Figure 3:**
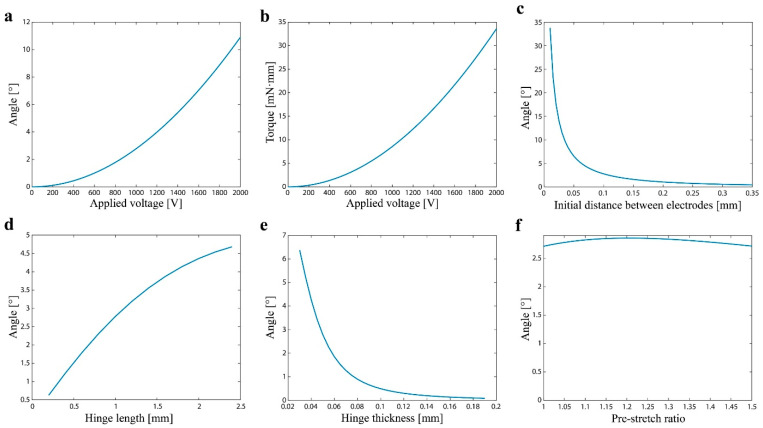
Typical output of the actuator model. (**a**) The tip angle and (**b**) torque as functions of the applied voltage. The tip angle as a function of the (**c**) initial distance between electrodes, (**d**) hinge length, (**e**) hinge thickness, and (**f**) pre-stretch ratio.

In this study, the model is implemented as a MATLAB code, where Equation (27) is numerically solved to obtain θ and τ for a given V. Figure 3 shows a typical output of the model. Both the tip angle θ and torque τ increase with the applied voltage V (Figure 3a,b). The size of the angle increases as the initial distance between the electrodes $d_{e0}$ decreases (Figure 3c). This is because the actuation of the DEA results from the Maxwell stress whose magnitude is proportional to the inverse of the distance between the electrodes. As indicated in Equation (26), the rotational spring constant of the hinge is proportional to the inverse of the length and the third power of the thickness. These change the rigidity of the hinge and, therefore, the output angle (Figure 3d,e). The model also suggests that the pre-stretch ratio of rolled DEAs has less impact on the actuation performance (Figure 3f). This may be because a large amount of pre-stretch stiffens the DEA in the stretched direction, which prevents actuation. Increasing the amount of pre-stretch slightly increases the actuation of the DEA by providing the pulling force from the other side of the DEA, but at some point, the effect of stiffening becomes dominant, and then the actuation is reduced.

## 3. Fabrication

Based on the analytical model, we designed the actuator. First, the initial thickness between the electrodes was set to 100 µm to facilitate the handling of DEAs during the fabrication. Then, the thickness and length of the hinge and the pre-stretch ratio were selected to achieve the target tip angle (in this study, 10°) at 2000 V. Under these specifications, we fabricated the actuator.

**Figure 4:**
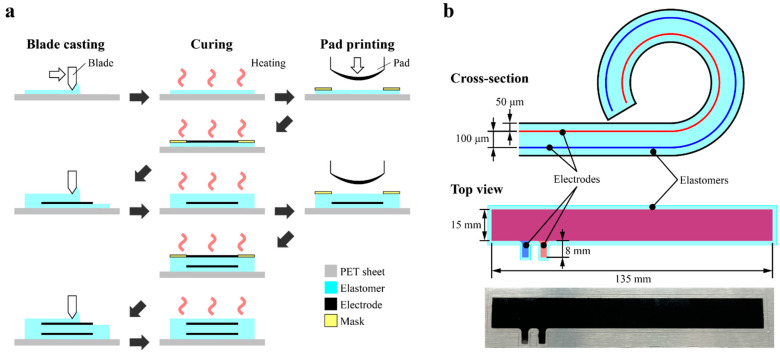
(**a**) Fabrication process of the actuator. (**b**) Structure and dimensions of the DEA part before rolling.

The actuator mainly consists of three parts: the rolled DEAs, the hinge mechanism containing the tip, and the backbone with base. The rolled DEA has a five-layer structure consisting of two electrodes and three dielectric elastomers, whose fabrication process is summarized in Figure 4a. The elastomer layer on the outside is to avoid contact between the electrodes of different polarities when rolled. Furthermore, these elastomers become a new dielectric layer when the inner and outer surfaces come into contact after rolling. Since the polarity of the electrode layers on both sides are different, an electric field is generated between the newly created dielectric layers, which functions as a DEA. For this reason, the thickness of the outer elastomeric layer was set to half the thickness of the one originally sandwiched by the electrodes, as can be seen in Figure 4b, so that the thickness of the dielectric layer of the DEA after rolling is equal. Moreover, since the dielectric layer is always outside after rolling, it acts as an electrical insulation allowing to use the actuator in water.

For the dielectric layers, a mixture of Ecoflex 00-30 (Smooth-On) and Sylgard 184 (Dow Corning) reported in another study [30] was used. The mass ratio of each silicone was Ecoflex 00-30: Sylgard 184: curing agent = 50:40:1. A mixture of carbon black, Ecoflex 00-30, and isooctane was used as the electrode material. The mass ratio of the electrode material was carbon black: Ecoflex 00-30: isooctane = 1:11:40. A film applicator (TQC Sheen B.V., AB4220) and a universal applicator (Zehntner, ZUA 2000) were used for casting the dielectric layer. A pad printer (Teca-Print, TPE 151) and a mask (Polypropylene film: 25 μm thick) prepared by laser processing were used for patterning the electrode layers. As summarized in Figure 4a, each layer was formed by curing in an oven at 80 °C for 1 h.

**Figure 5:**
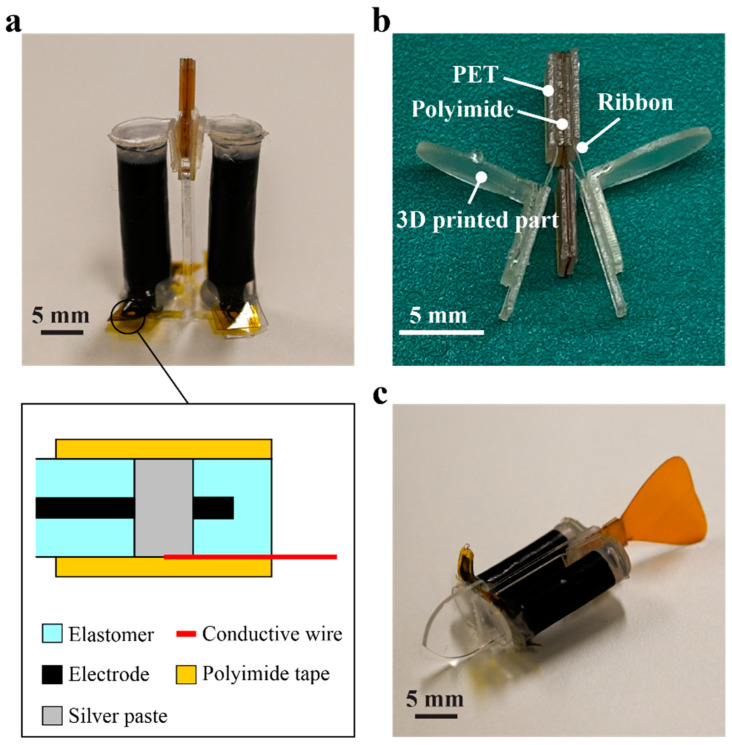
(**a**) Fabricated antagonistic actuator and its electrical connection. (**b**) Hinge mechanism. (**c**) Fabricated fish-type robot using the actuator.

After fabricating the five-layer DEA, the unnecessary parts were removed and the whole structure was rolled. During the rolling of DEA, a carbon rod with diameter of 1 mm was used as a core. Then, a PET sheet with a diameter of 7 mm was attached to both ends of the rolled DEA with silicone adhesive (Dow Corning, DOWSIL 734). A hole of 1 mm diameter was made on the exposed terminals of the DEA, and an electrical connection was established using a thin enamel wire (diameter 0.05 mm) and a silver epoxy (Gwent Electronic Materials, C60531D1). The fabricated rolled DEA, shown in Figure 5a, had a diameter of 6 mm, length of 21 mm, and mass of 0.6 g. Regarding the fabrication accuracy of the membranes, the total thickness of the five-layer DEA was designed to be 200 µm. The measured thickness of the six five-layer DEA samples was 208.1 ± 7.6 µm, slightly larger than the designed value. This may have resulted from the presence of electrodes. They had a thickness of a few µm, resulting in an error in the total thickness of the DEA, as such the value was higher.

The hinge mechanism of the actuator displayed in Figure 5b was fabricated by laminating several sheet materials: polyimide film (thickness 50 µm), PET film (thickness 250 μm), and double-sided tape. The ribbons made of polypropylene film (thickness 25 μm) were attached to the hinge mechanism and 3D printed parts using double-sided tape. The 3D printed parts were fabricated by Form 3 (Formlabs).

The backbone and base were fabricated from two parts that were laser cut from 1 mm thick acrylic sheets. These parts were connected using super glue. The height of the backbone determined the amount of pre-stretch applied to the DEAs. In this study, the height was set to 24 mm so that the pre-stretch was approximately 1.05.

The rolled DEAs, the hinge mechanism, and the backbone with base were assembled to form the antagonistic actuator. Masking tape was also applied to the contact points of the hinge mechanism and the backbone to reduce friction. The mass of the fabricated actuator was 1.7 g. The specification and model parameter of the actuator is summarized in Table 1.

## 4. Characterization

**Figure 6:**
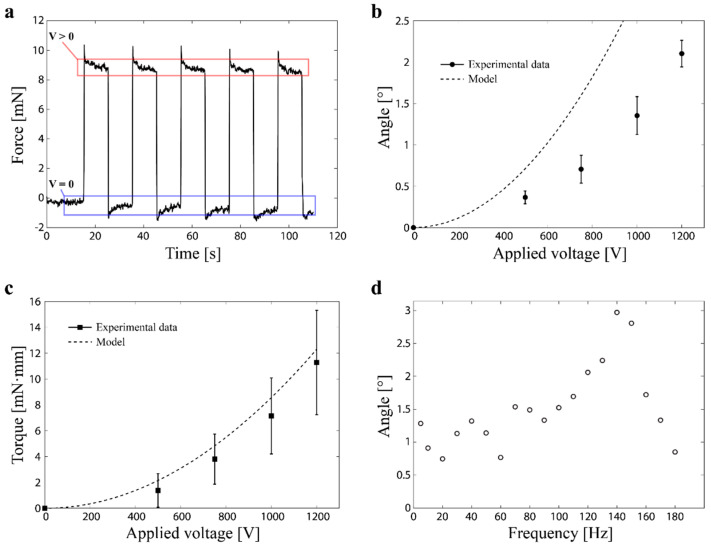
(**a**) Force output the actuator measured by a load cell. (**b**) Measured tip angle as a function of the applied voltage. (**c**) Measured torques as a function of the applied voltage. (**d**) Measured frequency response of the actuator (voltage 1000 V).

We measured both the angle and torque of the fabricated actuator as functions of the applied voltage as well as its frequency response. During these experiments, a high voltage power supply (HVPS v4b3 [31]) was used to actuate the device. For measuring the angle, a camera (Nikon, D7500) was used followed by image processing. In the torque measurement, a square wave voltage with frequency of 0.05 Hz was applied to the actuator and the values of a load cell, which was put on the tip was recorded. Figure 6a plots the output signal from the load cell, from which the force generated at the actuator tip was acquired, this was followed by multiplying the measurement position (i.e., moment arm) of 3.0 mm to obtain the torque. For the measurements of the output angle and torque, the applied voltage was set to 1200 V. In a preliminary test, we observed that the fabricated actuators tended to exhibit electrical breakdown within the voltage range of 1400–2000 V, even though the DEAs before the rolling process had withstood the applied voltage of 2000 V. This may have resulted from the tiny air voids existing in the rolled DEAs. During the rolling process, these air voids may be accidentally trapped between the surfaces of the DEA. They contribute to reducing the applicable voltage since the dielectric strength of air is ~3 V/µm [32]. To ensure a safe margin, we decided to use 1200 V to investigate the actuated angle and torque. For the frequency response, a square wave voltage of 1000 V was applied to the actuator while the motion was captured by the camera. In these experiments, given the symmetric structure of the actuator, only one side of DEA was activated.

The results are summarized in Figure 6b–d. The angle and torque increases as the applied voltage is increased. This suggests that the output of the actuator is voltage-controllable. The angle and torque at 1200 V take the value of 2.2° and 11.3 mN∙mm, respectively. From the results, it can be seen that the model well predicts the trend of the actuator output. The error between the model prediction and the experimental data may have resulted from several factors. Firstly, there is the friction between the DEAs and the hinge mechanism, which hinders the actuation. Secondly, the model does not consider the electrode layers that act as a passive element and reduce the amount of actuation. Thirdly, misalignment of the load cell may influence the measured force and, therefore, the torque. The measured frequency response indicates the presence of a resonance in the actuation, exhibiting an angle of 3.0° at the frequency of 140 Hz. The resonance actuation is useful in underwater robots that work by oscillation [12]. 

## 5. Swimming Demonstration

In order to confirm the applicability of the actuator for underwater robots, we fabricated a fish-type robot shown in Figure 5c. The materials used in the robot are identical to that of the actuator, while dimensions of the backbone and hinge are changed to form a head and caudal fin, respectively. The fact that the head and the caudal fin, both of which are necessary for realizing a swimming movement, can be obtained by simply changing their geometry is a unique feature of the actuator proposed in this study. The total length of the robot is 53 mm and weighs 2.2 g.

**Figure 7:**
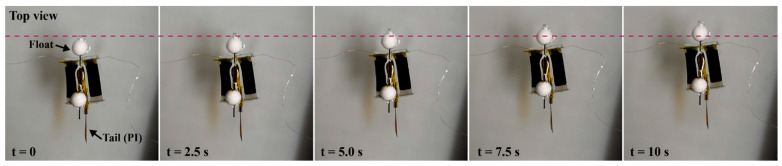
Sequence of the swimming of robot (voltage 1000 V, driving frequency 10 Hz).

Figure 7 displays a sequence of the robot swimming in water, where the two DEAs are activated alternately (see also Supplementary Video S1). As a result, a swimming speed of 0.9 mm/s was observed when a square wave voltage of 1000 V with frequency 10 Hz was applied. The swimming speed of the robot 0.9 mm/s, corresponding to 1.7 × 10^−2^ BL/s (BL: body length), is comparable to some of the DEA-based swimming robots (e.g., 1.9 mm/s (0.9 × 10^−2^ BL/s) [20] and 3.2 mm/s (1.0 × 10^−2^ BL/s) [17]). However, when compared to fast swimming robots (e.g., 64 mm/s (0.69 BL/s) [19] and 37.2 mm/s (0.25 BL/s) [12]), there is a significant difference. In short, the performance of the robot equipped with rolled DEAs is in the range of those of existing robots. It should be noted that those DEA-based robots are driven at relatively higher voltage, such as 5000 ([12]) and 10,000 V ([19]), when compared to that applied to our robot (1000 V). This suggests that the swimming performance of the robots, based on the antagonistic actuator with rolled DEAs, will be significantly increased once they are driven at higher voltages (i.e., higher electric fields). To do so, the dielectric elastomer used in the rolled DEAs could be replaced with an elastomeric material that has a higher dielectric strength. Moreover, optimization of the design of the robot (e.g., actuator dimensions and geometry of the fin) is expected to realize faster swimming movements. For this purpose, existing optimization techniques in soft robotics could be applied [33,34]. Nevertheless, we believe that our result already satisfies the objective of this study because our focus is to investigate and demonstrate the applicability of rolled DEAs for underwater robots through the development of an antagonistic actuator. 

## 6. Conclusions

In this study, to investigate the use of rolled DEAs for underwater robots, we proposed and developed an antagonistic actuator. The following results were obtained: Firstly, an analytical model was built, which guided the design of the actuator. Secondly, a fabrication process for this particular actuator was established. Thirdly, the actuator concept was demonstrated through characterization. Lastly, the actuator was successfully implemented in a swimming robot that showed locomotion in water and demonstrating the feasibility of using rolled DEAs in underwater robots. Future work should further characterize the actuator and robot, while also aiming to increase the output by improving the voltage tolerance of the DEA by reorganizing the fabrication process, modifying various aspects of the actuator design (such as the hinge mechanism), and enhancing the model accuracy by considering the presence of the electrode layers, followed by the development of other types of underwater robots.

## Figures and Tables

**Table 1:** Model parameter of the actuator.

Parameter	Value	Parameter	Value
Dimensions		Dimensions	
Rolled DEA		Fixing part	
Initial length $l_{\mathrm{DEA}0}$	21 mm	Thickness *w*	400 µm
Initial width $w_{\mathrm{DEA}0}$	141 mm	Material properties	
Initial thickness $h_{\mathrm{DEA}0}$	200 µm	DEA elastomer	
Initial inner diameter $r_{\mathrm{in}0}$	0.5 mm	Relative permittivity $\varepsilon_{r}$	2.8
DEA electrode		Material Constant $C_{1}$	0.0170 MPa
Initial length $l_{e0}$	15 mm	Material Constant $C_{2}$	0.00386 MPa
Initial width $w_{e0}$	135 mm	Material Constant $C_{3}$	−6.89 × 10^−5^ MPa
Initial width of margin $w_{m}$	2 mm	Hinge	
Initial distance $d_{e0}$	100 µm	Young’s modulus *E*_hinge_	3.3 GPa
Hinge		Ribbon	
Length $l_{\mathrm{hinge}}$	1 mm	Young’s modulus *E*_ribbon_	2.675 GPa
Width $w_{\mathrm{hinge}}$	5 mm	Other parameters	
Thickness $h_{\mathrm{hinge}}$	50 µm	Applied voltage *V*	0–1200 V
Ribbon		Pre-stretch ratio *λ*_1p_	1.05
Length $l_{\mathrm{ribbon}}$	2 mm	Permittivity of free space $\varepsilon_{0}$	8.85 × 10^−12^ F/m
Width $w_{\mathrm{hinge}}$	5 mm		
Thickness $h_{\mathrm{hinge}}$	25 µm		

## Data Availability

Not applicable.

## Supplementary Materials

The following are available online at https://www.mdpi.com/article/10.3390/polym14214549/s1, Video S1: Swimming of the fish-type underwater robot.
